# The Role of Hypertriglyceridemia in the Development of Atherosclerosis and Endothelial Dysfunction

**DOI:** 10.3390/nu6031236

**Published:** 2014-03-24

**Authors:** Saki Matsumoto, Nozomi Gotoh, Saori Hishinuma, Yohei Abe, Yoshimi Shimizu, Yumi Katano, Akira Ishihata

**Affiliations:** 1 Division of Theoretical Nursing and Pathophysiology, Yamagata University School of Medicine, 2-2-2 Iida-Nishi, Yamagata 990-9585, Japan; E-Mails: comedic2013@gmail.com (S.M.); nozomi.goto23@gmail.com (N.G.); saorihishinuma0818v@gmail.com (S.H.); yoshimizu1104@gmail.com (Y.S.), ykatano@med.id.yamagata-u.ac.jp (Y.K.); 2 Department of Pharmacy, Yamagata University Hospital, 2-2-2 Iida-Nishi, Yamagata 990-9585, Japan; E-Mail: abe@lsbm.org

**Keywords:** hypertriglyceridemia, atherosclerosis, visceral fat, liver, aorta, endothelium

## Abstract

A hereditary postprandial hypertriglyceridemic rabbit (PHT rabbit) is a new dyslipidemic model showing remarkably high plasma triglycerides with only limited elevation of plasma total cholesterol. In PHT rabbits, plasma triglyceride was markedly elevated postprandially compared with healthy Japanese white (JW) rabbits. In physiological experiments, the ring preparation of the thoracic aorta was suspended in an organ bath filled with modified Krebs-Henseleit solution, and the developed tension was recorded. Endothelial function was evaluated by acetylcholine-induced vasorelaxation in each preparation with intact endothelium. The acetylcholine-induced endothelium-dependent relaxation was diminished in PHT compared with JW rabbits, suggesting endothelial dysfunction in PHT rabbits. Histological examination was carried out in adipose tissue, liver and aorta. They were fixed in formaldehyde and embedded in paraffin. The tissues were sliced (4 μm) and stained using hematoxylin-eosin solution. In the adipose tissue, the visceral fat accumulated, and the size of adipose cells was enlarged in PHT rabbits. The liver of the PHT rabbit was fatty and degenerated. In aorta, increased intimal thickness was observed, suggesting the progression of atherosclerosis in the PHT rabbit. This study suggests the important role of postprandial hypertriglyceridemia in atherosclerosis. By using PHT rabbits, the effects of hypertriglyceridemia on health and diseases could be evaluated precisely.

## 1. Introduction

Dyslipidemia, especially hypercholesterolemia, is a risk factor for a variety of diseases such as hypertension, ischemic heart disease and cerebrovascular events. In those diseases, vascular atherosclerotic changes exist as a common pathophysiological mechanism. Atherosclerosis is considered to be related strongly to dyslipidemia such as hypercholesterolemia, where high plasma concentration of oxidized low density lipoprotein (LDL) may play a pivotal role in promoting vascular intimal thickening.

In addition to hypercholesterolemia, hypertriglyceridemia may be another factor for diseases. The strong relationship between serum triglyceride level and the mortality of ischemic coronary heart diseases was originally reported in the epidemiologic research carried out in 1985 as the Stockholm Prospective Study [1]. Then, several clinical studies have suggested that high triglyceride (TG) could become an independent risk factor for early development of atherosclerosis [2,3,4], and was correlated with other cardiovascular diseases [5,6,7]. For example, high concentration of serum non-fasting triglyceride was shown to increase the risk for myocardial infarction, ischemic heart disease, and death [8]. Therefore, it is recognized that treatment of hypertriglyceridemia is important for reducing atherosclerosis and its related diseases. Although a line of clinical studies support the predictive significance of triglyceride as an independent risk factor for coronary artery diseases, epidemiologic studies have several problems for analysis of data, for example, the serum triglyceride levels could be affected by diets, alcohol consumption, life style and the genetic background of the people, so these covariates must be adjusted for the interpretation of the data.

Because hypertriglyceridemia is reported to decrease the serum level of high-density lipoprotein (HDL) while it increases the remnant lipoproteins and small dense LDL, these effects would induce thrombogenesis, intimal proliferation and promote atherosclerosis [9]. One of the pathophysiological backgrounds for the increased risk for atherosclerosis and these diseases may be an endothelial inflammation and dysfunction [10]. In fact, it has been reported that the postprandial rapid rise in serum triglyceride levels after a high-fat meal was associated with transient endothelial dysfunction, as evaluated by the impairment in flow-mediated vasodilatation [11,12], and the endothelial dysfunction has been demonstrated to precede the formation of atherosclerotic lesion [13].

In spite of the implication from many clinical and physiological findings, the direct effect of triglyceride on the onset of atherosclerosis is not yet clarified from the pathological studies because triglyceride itself could not be detected in the atherosclerotic region, in contrast to the oxidized LDL. In addition, the problem that there were no adequate animal models of hypertriglyceridemia without hypercholesterolemia made it difficult to explore the precise role of triglyceride *in vivo*.

For the purpose of investigating the effect of triglyceride, a new animal model named PHT rabbit has been segregated in Yamagata University Animal Center to examine the link between triglyceride and cardiovascular diseases [14,15]. The preprandial triglyceride and cholesterol levels of PHT rabbits are within normal range. However, these rabbits show remarkably high levels of serum triglyceride after feeding, with little increase in serum cholesterol. The postprandial triglyceride levels continue to rise gradually up to 12 to 24 h after feeding, but the cholesterol levels change within the limited range [14,15]. It has been shown that young (around six-month-old) PHT rabbits have characteristics of central obesity by fat accumulation, but it is not yet known whether the long-term hypertriglyceridemia affects the physiological vascular function and promotion of atherosclerosis.

In this study, we investigated the role of triglyceride in the fat accumulation within the adipose tissue, in the fatty liver degeneration, and in atherosclerosis by using the postprandial hypertriglyceridemic PHT rabbits with different ages. The progression of atherosclerosis was evaluated by a histopathological method in combination with physiological examination of the age-related vascular endothelial dysfunction.

## 2. Materials and Methods

### 2.1. Animals and Drugs Used

Experiments were performed in accordance with the Guide for Care and Use of Laboratory Animals by the US National Institute of Health (NIH Publication No. 85-23, revised 1996 [16]) and under the regulation of the Animal Care Committee of Yamagata University School of Medicine. PHT rabbits were bred in the Laboratory Animal Center, Yamagata University School of Medicine. This study used male 12-months-old PHT rabbits (number of rabbits: five), male 33-months-old PHT rabbits (number of rabbits: five), and five male Japanese white (JW) rabbits (12-months-old, Shiraishi Laboratory Animals, Tokyo, Japan) for blood lipid assays and for pathophysiological examinations. In the physiological vessel relaxation experiments, additional rabbits (six-month-old JW; *n* = 4, six-month-old PHT; *n* = 4, 40-month-old PHT; *n* = 4) were used to examine the age-related changes in the endothelium-dependent relaxation of PHT rabbit (six-month-old PHT; *n* = 4, 40-month-old PHT; *n* = 4, and six-month-old JW rabbit as a normal control).

All the animals used for the experiments were bred and maintained under the conventional housing condition, and were clinically healthy. All animals were housed individually in the animal room controlled at 22 ± 2 °C, humidity at 55% ± 15% and a light: dark-cycle of 12 h:12 h (light on at 6:00). Each animal was fed standard rabbit chow (120 g/day, Labo R Grower; Nihon Nosan Kogyo, Tokyo, Japan) at 12:00 daily. The nutritional composition and energy value of the diets were as follows: crude protein, 17.1%; crude fat, 5.4%; crude fiber, 17.1%; crude ash, 9.6%; crude nitrogen-free extract, 43.5%; water 7.4%, and energy, 2087 kcal/kg. Water was supplied *ad libitum* by automatic watering system.

Body weights and abdominal circumference were measured under the anesthesia of pentobarbitone sodium (30 mg/kg). Then, rabbits were euthanatized with an intravenous overdose of pentobarbitone sodium (300 mg/kg) and exsanguination, and liver, thoracic aorta and visceral adipose tissues were isolated and weighed.

Acetylcholine chloride (Daiichi Pharmaceutical, Tokyo, Japan), pentobarbitone sodium and phenylephrine (Sigma-Aldrich, St. Louis, MO, USA) were used.

### 2.2. Measurement of Plasma Lipid Concentration

The rabbits were fed 120 g of standard diet daily at noon. For analysis of fasting triglyceride, the diet was withdrawn at 18 evening until next noon (fasting period: 18 h). Then rabbits were fed a standard diet again and started eating. The plasma triglyceride elevated gradually and reached maximum level after 15–18 h. Blood from each rabbit was collected via marginal ear artery after 18 h of fasting and at 18 h after the start of feeding. Then, blood was centrifuged at 3000 rpm (4 °C, 15 min) and supernatants were collected. Plasma concentration of triglyceride and cholesterol were measured by enzymatic methods using Triglyceride E- and Cholesterol E-test (Wako, Tokyo, Japan).

### 2.3. Histological Examination

Each tissue was fixed overnight in 10% formaldehyde at 4 °C. Tissues were embedded in paraffin and cut into 4 μm cross-sections. Microscopic examination was performed with hematoxylin-eosin stained sections to assess fatty changes in liver, adipose tissues and atherosclerotic changes in aorta.

### 2.4. Measurement of Vascular Relaxing and Contracting Function

**Figure 1:**
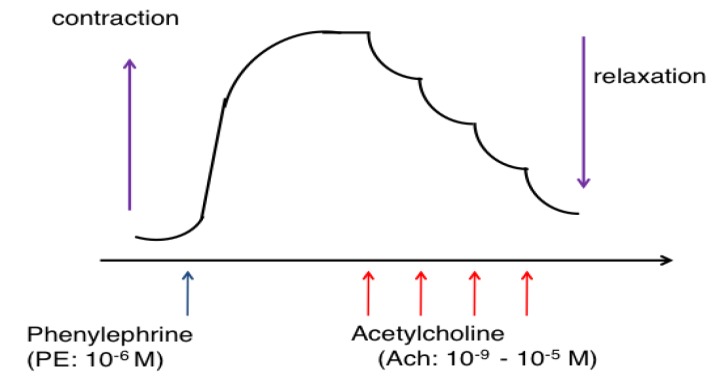
The method of measuring the response of vascular relaxation by acetylcholine in phenylephrine-precontracted aorta. Each preparation was precontracted with phenylephrine (1 μM) to reach maximal tension, then acetylcholine was added cumulatively to measure the relaxation response. In some experiments, the endothelium of the ring preparation was mechanically denuded to show the endothelium-dependent response.

Thoracic aortas were isolated and excess fat and connective tissues were removed. Vessels were cut into rings 2–3 mm long. In some preparations, endothelium was removed by gentle rubbing of the intimal surface with a forceps. Aortic ring preparations were suspended and incubated in organ baths containing modified Krebs-Henseleit solution gassed with 95% O_2_ and 5% CO_2_ (37 °C, pH 7.4). The solution contained 118 mM NaCl, 4.7 mM KCl, 24.9 mM NaHCO_3_, 1.18 mM MgSO_4_, 1.18 mM KH_2_PO_4_, 11.1 mM glucose, 1.8 mM CaCl_2_, and 0.057 mM ascorbic acid. The vascular developed tension was recorded by the isometric force transducer (7T-15-240, Orientec, Tokyo, Japan) for measurement of changes in the contractile force. The preparations were stretched to a resting tension of 2.0 g, and the solution was changed every 15 min. After an equilibration period of 1 h, each preparation was contracted with 66.7 mM KCl repeatedly until reproducible contraction was obtained. Then, preparations were precontracted with phenylephrine (1 μM) to reach maximal tension, and acetylcholine was added cumulatively to measure the relaxation response (Figure 1).

### 2.5. Statistics

All data are expressed as means ± SEM. Statistical analysis was performed by analysis of variance (ANOVA) with pairwise comparison by Tukey-Kramer test or by unpaired *t*-test. The difference was defined to be statistically significant when a *p*-value was less than 0.05.

## 3. Results

### 3.1. Effect of Hypertriglyceridemia on the Accumulation of Visceral Fat

The body weights of the PHT rabbits were 3.0 ± 0.1 kg (12-month-old) and 3.1 ± 0.1 kg (33-months-old), respectively. The weights of the visceral fats (per body weight) were 84.79 ± 3.59 (in 12-month-old PHT) and 63.75 ± 10.99 g/body weight (kg) (in 33-month-old PHT), respectively. The visceral fats were increased in PHT rabbits of both ages compared with 12-month-old Japanese white (JW) rabbits (Table 1). In addition, the liver weights of PHT rabbits (12- and 33-month-old PHT) also increased significantly compared to JW rabbits: the weights (g/body weight) were 39.97 ± 5.09 (in 12-month-old PHT), 27.37 ± 1.41 (in 33-month-old PHT) and 24.62 ± 1.45 (12-month-old JW), respectively.

**Table 1:** Comparison of the weights of visceral fat, liver and kidney.

Weight of Tissues (g/Body Weight: kg)	Normal Rabbit ^#^ (*n* = 5)	PHT: 12-Month-Old (*n* = 5)	PHT: 33-Month-Old (*n* = 5)
**Visceral Fat** **(Mesenteric Fat)**	39.73 ± 8.08	84.79 ± 3.59 *	63.75 ± 10.99 *
**Liver**	24.62 ± 1.45	39.97 ± 5.09 *	27.37 ± 1.41 *
**Kidney**	5.37 ± 0.16	7.44 ± 0.31 *	7.09 ± 0.23 *

**^#^** JW (12-month-old). PHT: postprandial hypertriglyceridemic rabbit. * *p* < 0.05 *vs.* JW.

### 3.2. Changes in the Concentration of Plasma Lipids in PHT Rabbits

**Figure 2:**
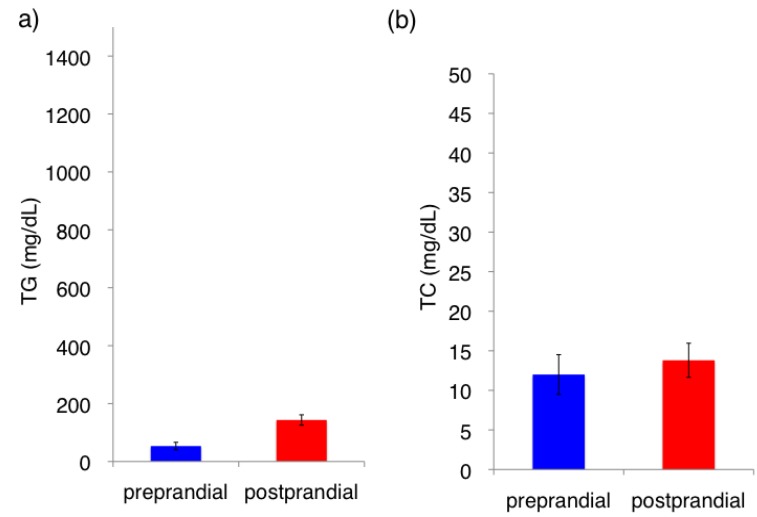
Plasma lipid concentrations in the normal JW rabbit before (preprandial; blue columns) and 18 h after feeding (postprandial; red columns). (**a**) Triglyceride (TG) (**b**) Total cholesterol (TC).

**Figure 3:**
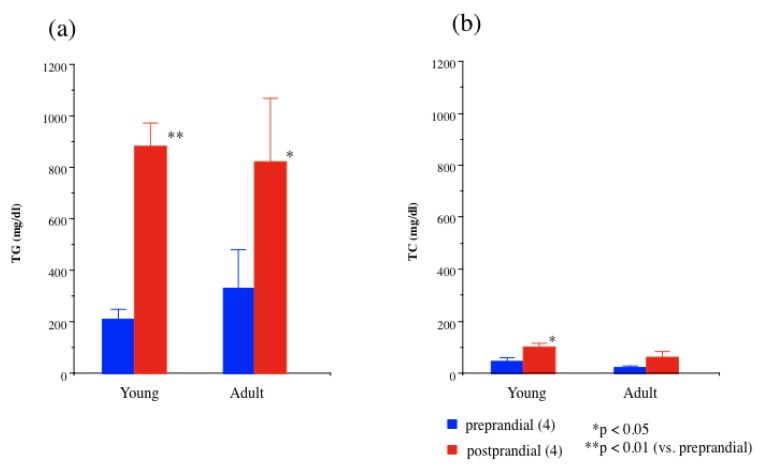
Plasma lipid concentrations in young and adult 33-months-old PHT rabbits before (preprandial; blue columns) and 18 h after feeding (postprandial; red columns). (**a**) Triglyceride (TG); (**b**) Total cholesterol (TC). Number of animals in parentheses.

The fasting (preprandial) triglyceride concentration in the normal Japanese White rabbit (JW) was <50 mg/dL (0.5 mmol/L) (Figure 2). In JW rabbit, the postprandial triglyceride level mildly increased (<200 mg/dL). In contrast, the fasting (preprandial) triglyceride concentration was 200–350 mg/dL (2.3–4.0 mmol/L) in PHT rabbits. However, postprandial (18 h after feeding) plasma triglyceride elevated to 800–1000 mg/dL (9.0–11.3 mmol/L) in 12-month-old and 33-month-old PHT rabbits, respectively. In contrast, little change in cholesterol concentration was observed 18 h after feeding (Figure 3).

### 3.3. Histological Examination of the Adipose Cells of Hypertriglyceridemic Rabbits

**Figure 4:**
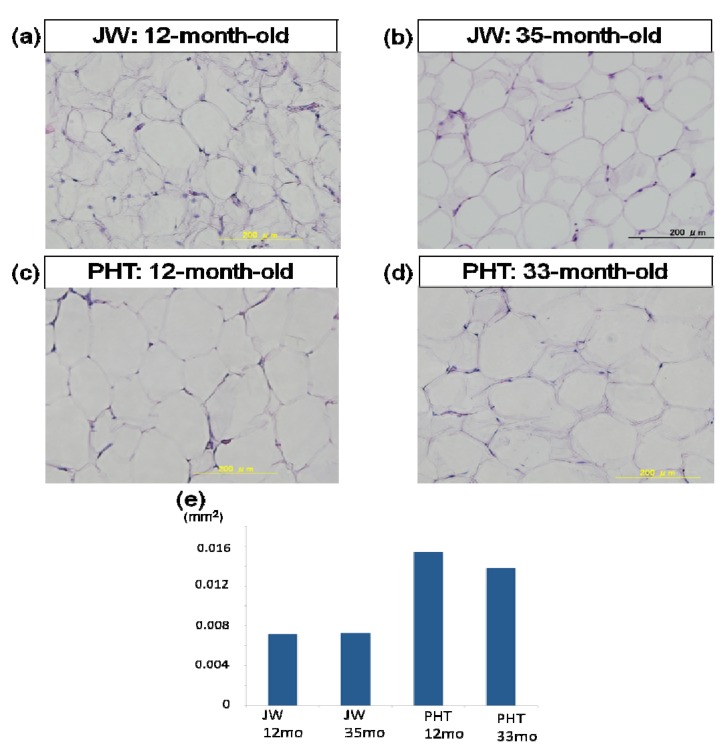
Effect of hypertriglyceridemia on the size of adipose cells. Histological examination of adipose cells (**a**–**d**) and size of adipose cells (**e**); Each tissue was fixed in formaldehyde and embedded in paraffin. They were sliced (4 μm) and stained with hematoxylin-eosin solution. (**a**) 12-month-old JW rabbit; (**b**) 35-month-old JW rabbit; (**c**) 12-month-old PHT rabbit; (**d**) 33-month-old PHT rabbit.

To clarify the morphologic changes in visceral fat of PHT rabbits, the histopathological examination was carried out (Figure 4). The adipose cells of PHT rabbits were filled with fats and the size was larger than that of JW rabbits. There were no remarkable changes in the size of adipose cells between 12-month-old and 33-month-old PHT rabbits.

In the mesenteric adipose tissue, the size of fat cell was measured. As shown in Figure 4e, the adipose cells were hypertrophied in PHT rabbits and lipid accumulation was increased in each cells.

### 3.4. Histological Examination of the Liver

**Figure 5:**
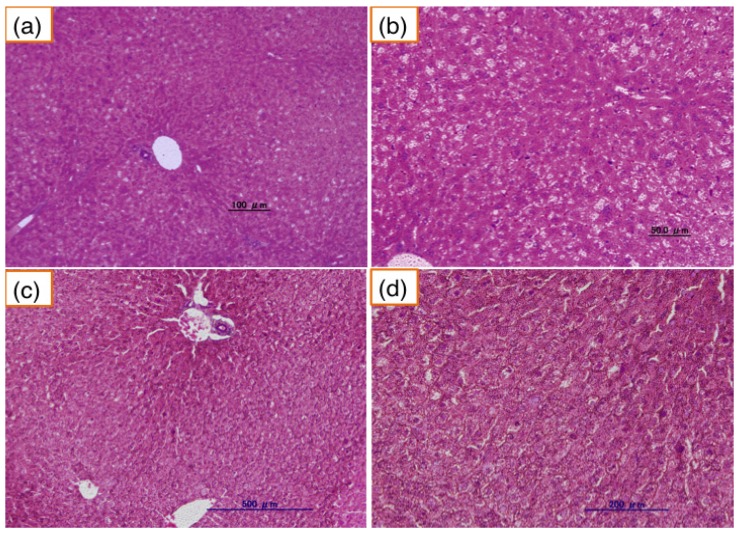
Liver of the normal JW rabbit. (**a**) and (**b**): 12-month-old JW rabbits. (**c**) and (**d**): 35-month-old JW rabbits. (**b**) and (**d**) are enlarged pictures of (**a**) and (**c**), respectively.

**Figure 6:**
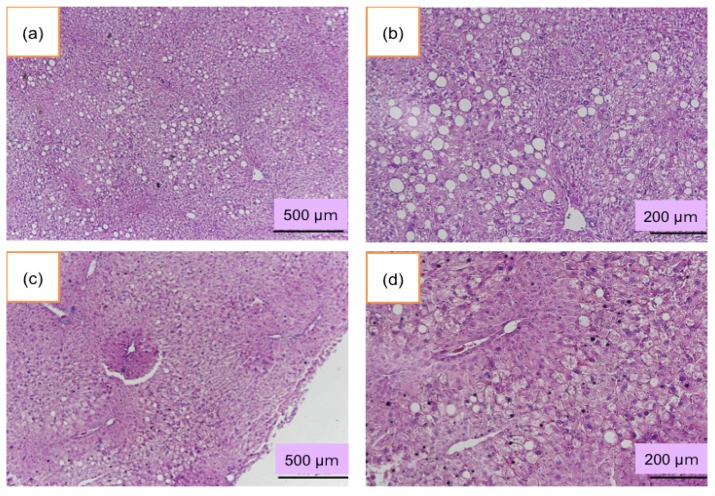
Lipid accumulation in PHT rabbit liver. (**a**) and (**b**): 12-month old PHT rabbits. (**c**) and (**d**): 33-month-old PHT rabbits. (**b**) and (**d**) are enlarged pictures of (**a**) and (**c**), respectively.

In the normal JW rabbit, liver did not show any pathological changes (Figure 5). In contrast, accumulation of fat in the liver increased in 12-month-old PHT rabbits (Figure 6). Hepatocytes were swelled and enlarged. The fatty changes were remarkable around the central vein. Some liver cells showed the ballooning degeneration of hepatocytes. Those ballooned hepatocytes were several times larger than normal liver cells, indicating steatosis. In 33-month-old PHT, liver cells especially around the central veins were also fatty degenerated. The accumulation of fat was demonstrated by the Oil-Red-O staining (data not shown). There were no inflammatory cells, and fibrotic changes were not observed.

### 3.5. Histological Examination of the Aorta of Hypertriglyceridemic Rabbits

**Figure 7:**
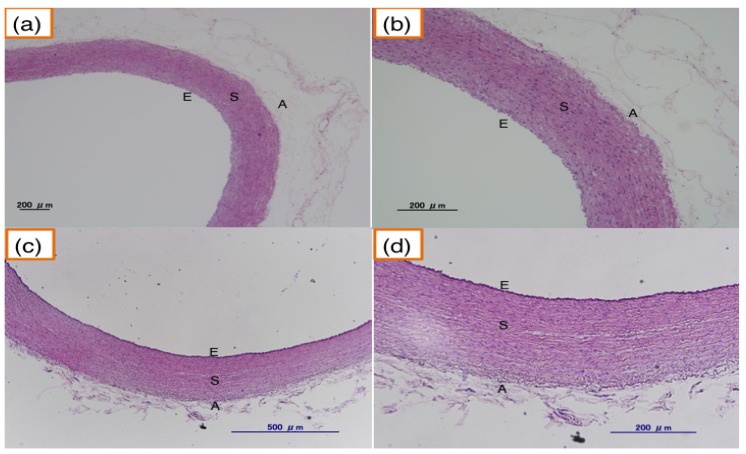
Aorta of the normal JW rabbit. (**a**,**b**) aorta from 12-month-old JW rabbit; (**c**,**d**) aorta from 35-month-old JW rabbit. E: endothelium, S: smooth muscle, A: adventitia, (**b**) and (**d**) are enlarged pictures of (**a**) and (**c**), respectively.

**Figure 8:**
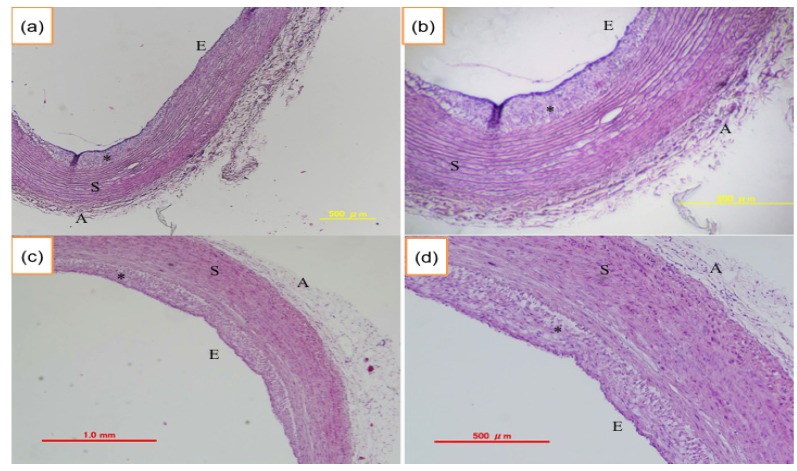
Atherosclerotic changes in PHT aorta. (**a**,**b**) aorta from 12-month-old PHT rabbit; (**c**,**d**) aorta from 33-month-old PHT rabbit. E: endothelium, S: smooth muscle, A: adventitia, * indicates atherosclerotic intimal thickening. (**b**) and (**d**) are enlarged pictures of (**a**) and (**c**), respectively.

JW rabbits (12-month-old, 35-month-old) did not show atherosclerotic lesions (Figure 7), while hypertriglyceridemic rabbits (12-month-old PHT) showed significant intimal thickening in aorta and the lesion grew and extended widely in 33-month-old PHT compared with 12-month-old PHT (Figure 8).

### 3.6. Endothelium-Dependent Relaxation in Hypertriglyceridemic Rabbits

**Figure 9:**
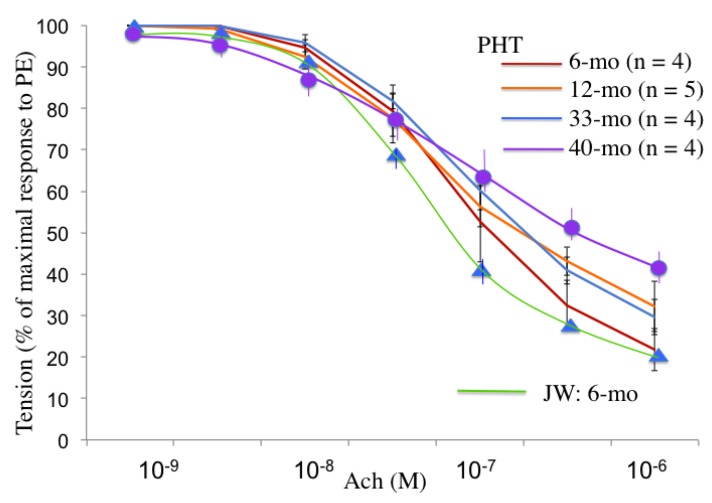
Age-related changes in the acetylcholine-induced endothelium-dependent relaxation of PHT aorta. The acetylcholine-induced relaxation in normal Japanese white rabbits is shown by the green line.

The atherosclerotic changes shown in Figure 8 were linked to the diminished acetylcholine-induced vasorelaxation. The vascular relaxing responses to acetylcholine were attenuated in the endothelium-intact aortic preparations of postprandial hypertriglyceridemic rabbits compared with those of JW rabbits. In the experiments with different ages of PHT rabbits, the acetylcholine-induced relaxation was less in 12- and 33-month-old than 6-month-old PHT. Furthermore, the vasorelaxation response in 40-month-old PHT rabbits was smaller than that of 33-month-old rabbits. The endothelial function evaluated by the acetylcholine-induced relaxation was decreased even in six-month-old PHT, and was affected age-dependently (Figure 9).

## 4. Discussion

In this study, the role of postprandial hypertriglyceridemia was investigated by using a newly segregated line of PHT rabbits. Although the preprandial triglyceride levels were low, plasma triglyceride concentrations reached to several times greater concentration of 800–1000 mg/dL after feeding, and the levels were not different between 12-month-old and 33-month-old PHT rabbits. In addition, the hypertriglyceridemia continued for a long period, at least up to 33-month-old. Previous studies showed that the triglyceride levels of normal 10- to 17-month-old Japanese white rabbits were 35.4 mg/dL in fasting and 62.8 mg/dL in postprandial conditions [15]. Previously it was reported that the retention of chylomicron remnants was caused by the delayed catabolism of exogenous lipids in the blood of PHT. In addition, the lipolytic enzyme lipoprotein lipase and hepatic triglyceride lipase were deficient and that the hepatic uptake of exogenous lipoproteins was delayed in the PHT rabbit [17]. In contrast, cholesterol levels in PHT rabbits were little changed even after feeding. Therefore, PHT rabbits that we used in this study have characteristics of triglyceride-selective dyslipidemia, and may be a useful model for studying postprandial hypertriglyceridemia.

### 4.1. Adipose Tissue in PHT Rabbit

The weights of visceral fats in PHT rabbits were increased in 12-month-old, compared with 6-month-old PHT rabbits as well as that of JW rabbits, and the accumulation of fat was not different between 12-month-old and 33-month-old rabbits (Table 1). In accordance with our present data, a previous report showed the increased accumulation of visceral fat of 8-16-month-old PHT rabbits compared with that of Japanese white rabbits [14]. We also demonstrated histologically that the fat cells of mesenteric adipose tissues were enlarged compared with those of JW rabbits. The swelling of mesenteric adipose cells remained until at least 33-month-old PHT rabbits. It is known that adipocytes can produce adipocytokines such as adiponectin and leptin in normal physiological conditions. The serum adiponectin has anti-atherogenic and anti-inflammatory effects, and leptin has lipolytic and appetite suppressing effects [18]. However, in pathologic conditions, the function of adipocytes would be altered. Indeed, several studies have demonstrated that the inverse relationship between plasma adiponectin levels and inflammatory markers such as C-reactive protein. Also, there was a correlation between postprandial triglyceride and interleukin-6 concentrations in diabetic patients. The anti-inflammatory properties may have a pivotal role in protecting against metabolic diseases such as atherosclerosis. However, it has been shown that adiponectin production in adipocytes from obese people decreased compared with that of non-obese men. Therefore, it is implicated that PHT rabbit may have a low adiponectin levels compared with JW rabbits. Actually, Ito *et al*. [19] reported that adiponectin mRNA was downregulated in PHT rabbit. The accumulation of visceral fat and hypertrophy of adipocytes may decrease the production of adiponectin and leptin, while the production of cytokines such as TNF-α, PAI-1, angiotensinogen and resistin may be increased. TNF-α causes insulin resistance and inflammation response, and PAI-1 promotes thrombosis and inflammation. In this study, PHT rabbits showed visceral fat accumulation and adipocytes were hypertrophied until 33-month-old. Therefore, postprandial hypertriglyceridemia and visceral obesity may be responsible for the early onset of atherosclerosis, and it is speculated that changes in the production of adipocytokines such as adiponectin, TNF-α, resistin and PAI-1 would be responsible for the progression of atherosclerosis in PHT rabbits.

In PHT liver, there was a remarkable fatty degeneration in the hepatocyte around the central veins. It is known that fatty liver is caused by obesity, especially by visceral fat accumulation [20]. Recently, the fatty degeneration of the liver is reported to be a risk for non-alcoholic steatohepatitis, which may cause cirrhosis and cancer. However, in our present study, the liver of PHT rabbits showed neither inflammatory cells nor fibrous changes, suggesting that the liver was in a simple fatty liver degeneration.

### 4.2. Endothelial Dysfunction and Atherosclerosis

In this study, the progression of atherosclerosis was evaluated by physiological as well as histopathological experiments. The intimal thickening of the thoracic aorta was detected most frequently in 33-month-old PHT rabbits, and the region extended widely. These observations indicate that the progression of atherosclerosis in PHT rabbits is age-dependent.

Whether triglyceride can trigger the atherosclerosis is controversial. Triglyceride is water-insoluble and delivered in blood vessels as lipoprotein-bound form. Hypertriglyceridemia is usually accompanied by high levels of chylomicron and VLDL. Chylomicron is metabolized and the chylomicron remnant is suggested to be able to promote atherosclerosis [21]. Because chylomicron and VLDL remnant is a small particle, it may infiltrate into sub-endothelial space and be trapped in the arterial wall to make atherosclerotic plaques [22,23]. In fact, Kawai *et al*. [15] reported that the levels of chylomicron and VLDL in PHT rabbits were higher than those of Japanese white rabbits. Another possible reason for the early progression of atherosclerosis in hypertriglyceridemia may be the presence of small dense LDL. In hypertriglyceridemia, LDL reportedly becomes a relatively small and dense particle called small dense LDL. Because the metabolism of small dense LDL is slow and it may exist in the plasma for a long time, it can easily contact with endothelial cells and infiltrate into sub-endothelium, and is oxidized and phagocytosed by macrophages. It may cause endothelial dysfunction at the same time.

In human and WHHL rabbit, apoproteins B48 and B100-containing lipoptoreins were detected in the aortic intimal lesions and implicated to promote atherosclerosis [24,25]. These apoproteins could be involved in the progression of atherosclerosis in PHT. In fact, a previous report examined the kinetics of various apolipoproteins in PHT rabbit to elucidate the lipid metabolism in PHT. It was shown that apoprotein E, which is necessary for the uptake of chylomicron remnants by the liver, was increased postprandially in PHT [17]. In the SDS-PAGE experiments, apolipoprotein B100 was detected in the PHT rabbits before feeding, and apolipoproteins B100 and B48 were both detected 15 h after feeding. In contrast, in the JW rabbits, neither apolipoprotein B100 nor apolipoprotein B48 was detected before or after feeding [17]. Because apolipoprotein B48 was detected only in PHT after feeding, they suggested that exogenous lipoproteins remained unmetabolized after feeding in PHT [17].

Mero *et al.* reported the coronary angiography data suggesting that small chylomicron remnants were implicated in the progression of coronary artery diseases in patients with type 2 diabetes mellitus [26]. In accordance with our present results, the association of postprandial hypertriglyceridemia and carotid intimal and medial thickness has been reported in patients with type 2 diabetes [27].

Endothelial dysfunction was evaluated by a physiological method using isolated thoracic aorta. The endothelium-dependent relaxation by acetylcholine occurred concentration-dependently in aortic ring preparations. The relaxation response was diminished in 12-, 33- and 40-month-old PHT rabbits compared with that of 6-month-old PHT rabbits, suggesting the endothelial dysfunction in older PHT rabbits, probably due to the decreased production of nitric oxide which plays an important role in vascular relaxation, inhibition of platelet adhesion and aggregation. These results are consistent with the histopathological changes observed in PHT aorta. Together with these results, it could be implicated that endothelial cells might be injured and atherosclerosis could be accelerated in hypertriglyceridemia. In addition, the atherosclerotic vessels would induce hypertension. In fact, Fukuda *et al.*, showed that blood pressure measured by a telemetry system was significantly higher in PHT than JW rabbits [15,28].

## 5. Conclusions

This study showed that the marked elevation of postprandial plasma triglyceride, visceral fat accumulation, fatty degeneration of liver, early atherosclerotic intimal thickening and rapid onset of vascular dysfunction in endothelial cells in a hypertriglyceridemic model of PHT rabbits. These results suggest that postprandial hypertriglyceridemia may be an important risk factor for promoting atherosclerosis.
